# Open, trusting relationships underpin safety in rural maternity a hermeneutic phenomenology study

**DOI:** 10.1186/s12884-016-1164-9

**Published:** 2016-11-24

**Authors:** Susan Crowther, Elizabeth Smythe

**Affiliations:** 1 School of Nursing and Midwifery, Robert Gordon University, Garthdee Road, Aberdeen, Scotland, UK; 2 Auckland University of Technology, Auckland, New Zealand

**Keywords:** Rural maternity, Midwives, GPs, Relationships, Discord, Safety, Phenomenology, New Zealand

## Abstract

**Background:**

There are interwoven personal, professional and organisational relationships to be navigated in maternity in all regions. In rural regions relationships are integral to safe maternity care. Yet there is a paucity of research on how relationships influence safety and nurture satisfying experiences for rural maternity care providers and mothers and families in these regions. This paper draws attention to how these relationships matter.

**Methods:**

This research is informed by hermeneutic phenomenology drawing on Heidegger and Gadamer. Thirteen participants were recruited via purposeful sampling and asked to share their experiences of rural maternity care in recorded unstructured in-depth interviews. Participants were women and health care providers living and working in rural regions. Recordings were transcribed and data interpretively analysed until a plausible and trustworthy thematic pattern emerged.

**Results:**

Throughout the data the relational nature of rural living surfaced as an interweaving tapestry of connectivity. Relationships in rural maternity are revealed in myriad ways: for some optimal relationships, for others feeling isolated, living with discord and professional disharmony. Professional misunderstandings undermine relationships. Rural maternity can become unsustainable and unsettling when relationships break down leading to unsafeness.

**Conclusions:**

This study reveals how relationships are an important and vital aspect to the lived-experience of rural maternity care. Relationships are founded on mutual understanding and attuned to trust matter. These relationships are forged over time and keep childbirth safe and enable maternity care providers to work sustainably. Yet hidden unspoken pre-understandings of individuals and groups build tension in relationships leading to discord. Trust builds healthy rural communities of practice within which everyone can flourish, feel accepted, supported and safe. This is facilitated by collaborative learning activities and open respectful communication founded on what matters most (safe positive childbirth) whilst appreciating and acknowledging professional and personal differences.

## Background

Rural communities globally have unique and specific needs which come to the fore in the experience of childbirth. The clinical significance of maintaining sustainable maternity services in these communities has been demonstrated [1]. For the woman and her family and the health professionals who deliver rural maternity care there are interwoven personal, professional and organisational relationships to be navigated. These relationships are integral to maternity care [2–6] and are particularly pertinent in rural maternity care provision [7, 8]. This paper reports on an aspect of a larger study examining rural maternity experiences from a variety of professional and user experiences in New Zealand [9]. The theme of relationality resonated throughout the data of this study as a key component of safe care and is the focus of this paper. The study was undertaken within a well-resourced and established healthcare infrastructure. Review and discussion of the literature is therefore focused on similar regions.

New Zealand has a unique maternity system based upon continuity of care. Most care is provided by Lead Maternity Care providers (LMCs) who are mainly self-employed midwives with some GPs and obstetricians taking on this role. The Ministry of Health directly funds LMCs, who are either a midwife or a doctor (e.g. GPs) with obstetric qualifications [10]. Over the years, most GPs have discontinued providing maternity services leaving LMC midwives as the main providers [11]. When the LMC is not available rural based New Zealand GPs are called upon to assist with maternity care and emergencies not required in urban areas [12]. Women choose from available LMCs in their region for pregnancy, birth and postnatal care; although choice is often limited to one care provider in a rural situation. Such care is free to citizens and residents of New Zealand. The maternity service also employs core midwives who provide hospital based midwifery services to women when they are inpatients. LMCs are responsible for coordinating women’s maternity health care from early pregnancy until 6 weeks postnatally, this includes full intrapartum care [13]. They provide primary services as well as provide care to women in secondary services if they are booked with them.

The LMC through ongoing assessments will decide in partnership with the woman if referral or not is required to other services. For example if a woman and infant have straightforward antenatal, birth and postnatal experience then the LMC makes no referrals to medical colleagues. LMC midwives work across the full scope of their practice within referral guidelines and are accountable for the decisions they make. The referral guidelines for consultation with obstetric and related medical services list the conditions for referring women or their infants to specialists or other primary caregivers. The guidelines are evidenced based and agreed by midwives, obstetricians, other health care specialists and consumers [14]. These guidelines are reviewed and updated at least every 5 years. The delivery of maternity services in New Zealand is underpinned by the requirements of the national Section 88 Maternity Notice regulated by the Ministry of Health [10]. These institutions oversee the service and payments of primary maternity care provision.

Rural New Zealand communities are diverse with small populations living over large geographical areas [15]. Defining what is rural is complex and there remains no consensus on what constitutes rural health care [16, 17]. It has been suggested that researchers define and name rural as pertinent to the project and select or formulate an appropriate definition [18]. Informed by personal experiences, discussion with rural health care providers and examination of national New Zealand policy documents and international definitions the definition of remote rural for this study is:

*A locality in which experiences of maternity occurs 60 min or more by road* (*in optimal weather conditions*) *from secondary hospital services as determined by those that live and*/*or work in these regions who have local knowledge of actual lived travel times*.

Most maternity care in these regions is provided by rural LMC midwives working in isolation [11]. The skill set and qualities required for maternity care providers in rural areas has been shown to be similar to urban colleagues; yet significantly different. Being confident and making autonomous decisions and knowing when to call for back-up are essential [7, 19–24]. An ability to work inter-professionally and collaboratively has also been highlighted as essential in rural practice [7, 20, 22]. It is apparent that these skills require relationships inter and intra-professionally and collegial communication pathways with necessary supplementary skills and resources. Some of these relationships may be within community and ideally face-to-face whilst others are at a faraway distance where the rural midwife may be unknown to the person on the other end of the phone. It can be surmised that the experience of providing and receiving maternity care in rural areas is very different to urban areas of New Zealand. However there is a paucity of research on how relationships influence safety, nurture satisfying practice for rural maternity care providers and what promotes positive experiences for mothers and families in rural regions. This paper reports on a study that reveals how relationships are an important and vital aspect to the lived-experience of rural maternity care.

## Methods

The rationale for using a qualitative methodology for this study was based on the need to gather data that provided in-depth and rich stories of the lived experience of rural maternity. Bearing witness to the stories in these regions has value providing an opportunity to review, reflect and inspire new thinking.

Interpretive hermeneutic phenomenology guided the approach to data collection. Unstructured in-depth interviews with women and rural health care providers were conducted making it possible to reveal further understandings of the meaning of rural maternity experiences. The focus of this approach is the surfacing of meaning from lived experience descriptions bringing insights that gesture to our shared humanness [25]. The ontological literature provides an avenue into in-depth exploration of ‘being-with-other’ and ‘being-in-the world’ as found in the writings of Heidegger. His interpretation of ‘being’ gives the foundation to further interpretation [26]. Further, the work of Gadamer is drawn upon to show how language itself can ‘speak us’ and how the text itself interacts with the investigator, through the ‘fusion of horizons’ or perspectives [27, 28].

In-depth interviews of one to 2 hours with families, midwives, GPs and ambulance staff in a range of remote/rural areas in New Zealand were undertaken. A total of 13 interviews were conducted following signed consent in a place suitable to participants in the regions in which they lived. Participants were asked to share their experiences of rural maternity care. Although there were prior indicative questions, participants were able to take the discussion in directions that were important and significant to them whilst ensuring that key research questions were addressed. The method was largely informed by van Manen [25, 29]. Interviews were transcribed and analysed using a phenomenological approach for themes and patterns that highlighted areas that spoke to us as being important. Lived experienced descriptions from these manuscripts were edited into crafted stories and then analysed [30, 31].

### Pre-understandings

All meanings are informed by fore-structures of understanding that are culturally, socially and environmentally constructed. It is important to reveal our pre-understandings and judgements as they shape the questions brought to the study and interpretation that followed [28].

Susan was the principle investigator. The majority of her practice career had been in the primary sector with a particular focus on rural midwifery. She has worked in several remote rural locations as a midwife in Europe, developing countries and in New Zealand. She was interviewed by her co-author, Liz, prior to the start of this study in order to highlight her own pre-understandings and judgements. Liz, long ago, had an experience of practising in a remote mission hospital in the developing world, and has visited many remote communities. They shared an understanding of birth in situations where there is no immediate support. Susan’s own stories of practice in rural New Zealand were rich in joy, but the exhaustion, fear and burden shone through. We came to this study both in awe of women’s ability to birth ‘naturally’ but at the same time very mindful that things can go wrong, quickly. We believe in the ideal world women have a right to give birth close to their own community.

### Recruitment of participants

Purposeful sampling by word of mouth was used to recruit study participants from the South and North islands of New Zealand. Professional and social networks were used to initiate interest. Participants were recruited by a process of snowballing. A letter of invitation and information sheet was sent once a potential participant had signalled an interest by telephone, email or a third person mutual contact. Care was taken to prevent coercion. Non response to the formal invitation was an easy way for people to opt out of this study. Participants not included were women and/or partners experiencing some degree of depression following birth, health care practitioners involved in complaint proceedings, present students of AUT University (where the principle investigator was working as a senior lecturer) and no one under Susan’s professional midwife care at the time of the study.

All 13 participants are volunteers. They all have an interest in the phenomenon. They all live and work within different rural regions of New Zealand and come from a variety of cultural, professional and social backgrounds:

- 3 × Mothers
- 6 × Midwives: Registered practising midwives
- 3 × Ambulance crew (paid and unpaid)
- 1 × General Practitioner Obstetrician

Crafted stories from the raw transcriptions were returned to participants for their critique. Participants were thus able to ensure that the crafted stories reflected their experiences as narrated in the interview. Due to the complexity and sensitive nature of data gathered in small population regions, constant efforts were made to ensure anonymity as much as possible. Names, places and institution identifying features were removed from the crafted stories. Several participants wanted details of stories removed or changed to ensure they would not be identified. This did not alter experiential meaning that surfaced from the data or diminish the emergent themes found in the data overall. Each participant was provided an agreed pseudonym. All responses were treated confidentially to protect the privacy of participants. Transcriptions were conducted by a professional transcriber who signed a confidentiality form.

### Interpretive analysis

Analysis was done using an interpretive hermeneutic phenomenological process involving a process of writing and re-writing in which stories were crafted from the raw interview data and then analysed for meaning [32]. The interpretive analysis of the stories required a dialectic movement between all the stories and the detail of individual stories involving cycles of thinking-writing-reading until a plausible thematic argument emerged [25, 29]. Through moving between parts and whole of crafted stories, and all stories together, themes emerged and new horizons of understanding surfaced as clusters of stories revealed commonalties and resonant qualities. Reading and re-reading the whole captured the core significance bringing to light the phenomenon of New Zealand rural maternity experience. Exploration of historic and contemporary New Zealand, and international literature from midwifery and other relevant disciplines, was incorporated within the project in order to provide context. The purpose of analysis is to provide rich uncovering of the phenomenon in order to provoke further thinking and call to action. Phenomenology does not claim to reveal everything there is to know about phenomenon; this is not possible.

Ethics approval was obtained prior to recruitment and data collection. Ethics approval was gained through AUTEC reference No. 15/18 on 9th February 2015. Approval included a researcher personal safety protocol.

## Results

Throughout the data the relational nature of rural living surfaced.

### Showcasing optimal relationships

Michelle (midwife) describes how relationships matter and engenders supportive community spirit.

*It*’*s nice being in a small supportive community. It*’*s a great job being a rural midwife. There are niggly frustrating things but at least we*’*ve got each other*; *maintaining a good group*, *having a good relationship and looking after one another. This area has great support for the mums and babies. Women around here know each other and are all very supportive of one another*. (*LMC midwife*, *Michelle*)

Michelle explains how there is often someone to help when needed; even if that help is not immediately to hand in terms of distance and time. There is an emotional connectedness born of sharing the same rural life style and need to remain associated with each other in some way or other. Midwives find themselves thrown into the lives of the community in their work and close relationships are forged. Everyone is committed to supporting whatever it takes to make childbirth a positive experience. Caroline explains how families and LMC providers become near both physically and emotionally.

*She* [*a mother*] *is making the decisions she needs for her family. I*’*m here just walking and guiding and sitting. It is fantastic just to see the baby in her arms*; *it*’*s brilliant. That*’*s what I do this for. I see her work so hard and see her accept it*; *then I see the first encounter when she and her partner go* ‘*wow*’. *It is a privilege to be invited into these moments. It*’*s so intimate and close*. (*LMC midwife*, *Caroline*)

This quality of delight in giving parents such a good childbirth experience builds over the pregnancy, culminating with a deep sense of awe when the midwife has the privilege of being invited into the intimate space of the new family. There is a sense that such moments make the hugeness of the job (being on call for long hours, travelling long distances) worthwhile. Deepa (GP) shows how relationships over time matter in her practice.

*I*’*ve currently got a lovely lady who*’*s got incredible anxiety because of what*’*s happened before in her pregnancies. It*’*s becom*i*ng more of a mental health issue. I can keep contact as her GP*. I c*an get buy in from the whole family. Having GP input in maternity is integral in the rural area. Continuity is really important. It*’*s bizarre to lose my patient for 9 months and have no contact. Patients enjoy continuity*. (*GPO*, *Deepa*)

Continuity matters to those involved in maternity care provision; whoever they are. In urban New Zealand it is unusual to have a General Practitioner involved in routine maternity care. That has become the domain of midwives. Deepa’s story shows how in the rural setting, it works for her to stay closely involved with clients. Familiarity between care providers and families nurtures such trust, enables safety and reduces fear. It appears that this trust facilitates positive experiences and creates potential for safer rural childbirth.

There is empirical evidence to support how midwife continuity of care promotes calmness and reduces intervention [3, 33, 34]. Knowing care providers within the community over time keeps childbirth safer; the knowing born of continuity seemingly has a wraparound preventative quality. Maintaining continuity in the rural regions is not the sole remit of midwives. Continuity for Deepa (GPO) and this mother speaks powerfully of continuity which transcends disciplines.

### Feeling isolated

Feelings of isolation and unrest are felt when physically alone, away from the familiarity of home, family, friends and health care providers whom one feels safe to call upon for help and advice. Feeling separated from others is unsettling for Sarah (mother) when she is admitted to the urban hospital.

*We did not know a soul in the town*, *never been there before. We had no visitors*, *everyone we knew was so far away it was quite isol*a*ting. We had one friend of a friend from around here pop in which was quite nice*. (*Mother*, *Sarah*)

Being away from home and everyday familiarity is challenging for Sarah. It is this sense of alienation that drives communities to retain birthing services close at hand, yet when there is an issue the woman may have no choice but to transfer to a place where she knows no one and is unknown. This feeling of isolation is also felt by Marie (midwife) professionally and personally.

*It is very different working here. The first year I had uncomfortable feelings of feeling very isolated. In secondary care I can just ring a bell and a whole heap of people come. I just feel really alone clinically and also lonely socially because in a bigger unit I might be busy passing people in the corridor*, *catching up with people at tea break. Here I can actually do a whole shift without seeing anybody else apart from clients that I*’*m looking after. I*’*m quite a social person and I really miss that social interaction. Having other health practitioners that I can either run ideas past or in case of emergencies depend on their help*; *having others around is like a security blanket feeling. I realise that I have got all skills and knowledge to cope with emergencies here but I still feel alone*. (*Rural facility core midwife*, *Marie*)

Professional isolation is unsustainable and unsettling for Marie. Working alone, feeling isolated, brings into relief how relationships are vital. Sustainable practice requires reciprocal relationships [35]. Marie has learnt to cope with the difference in rural maternity care provision; yet there is a sense that professional isolation can be unsafe in both a practical sense and emotional wellbeing.

### Living with discord

A disturbing finding within the study was practitioners who remained ‘alone’ in situations where there were others on hand who would have willingly helped. When New Zealand midwives won autonomy to practice in their own right [36] midwives stepped forward and General Practitioners stepped back. The funding mechanisms of practice and the political climate where professional territory was fiercely guarded discouraged collegial relationships. Deepa’s story reveals the consequences of a lack of relationship between the local midwife and doctor:

*There was a patient sitting in an ambulance on the rural hospital grounds. I didn*’*t realise why a chopper was coming. It wasn*’*t until the ambulance control rang the hospital to say the chopper*’*s 10 min away for your PPH that I realised it was a PPH. I was aghast because when we looked out the back doors suddenly there was an ambulance with a woman inside who did not have an IV drip in having a PPH and just the LMC midwife in the ambulance. We do hold O negative blood at the rural hospital so it was highly appropriate that the woman was provided medical resus treatment and stabilised. I felt embarrassed as an institution that a midwife had arrived with somebody in such extremeness and I was not involved. What was a barrier to that*? *I don*’*t know. Team work in maternity in the rural areas is vital. It is a cultural issue between doctors and midwives. However if you have doctors that don*’*t have any obstetric knowledge at all then they*’*re going to stay well away leaving a gap in the rural community. Midwives working with doctors in rural areas would be helpful*. (*GPO*, *Deepa*)

The discord within this story points to conflict and resentment. Deepa describes how professional isolation can lead to safety being compromised. Her story reflects the voices of GPOs interviewed in Miller et al.’s 2013 study who found that GPOs were concerned that there was a paucity of reciprocal professional assistance leading to potential for poor outcomes [37]. This was especially true in areas where there was a shortage of midwives. The GPOs in Miller’s New Zealand study, like Deepa, were concerned that rural LMC midwives end up working without any support from a professional colleague, be it another midwife or a general practitioner. The concern in Deepa’s story is that she was ‘there’ well able to provide valuable clinical skills but was not given that opportunity. The recent Miller and Preston studies argue for rural GPs to retain obstetric skills and have shared care with midwives in rural regions [37, 38]. Yet differences in philosophies of care creates potential for discord.

Michelle (LMC midwife) speaks about how differing values between midwives and GPs can be magnified in these small communities. She clearly recognises the importance of building relationships with her medical colleagues but does not find that reciprocated:

*When we set up this practice*, *I said*, “*look it*’*s important to develop a relationship with the GPs. Often we hit a bit of a glitch and then we have to have a meeting and say look what*’*s going on and have we upset you in some way*, *what*’*s the problem*? *Is there a communication issue*, *you know because we give a lot to them. We can go and see them before we have a real negative kind of relationship*”. *We try and communicate*, *we*’*ve tried. The communication efforts are not reciprocal. For example when a new midwife comes to the area she makes herself known to the GPs*, *if a new GP comes to this area we are not informed. The relationship could be better. I had great relationships with GPs in the past in community*; *it really is weird here* – *the GPs hold you at arm*’*s length. It*’*s like* ‘*we don*’*t really know you*, *we don*’*t really want to know you*, *and we don*’*t want to know anything about obstetrics because we*’*re not doing it. But we need you*’. *GPs expect you to come now when they call. It would be better if this was more reciprocal*. (*LMC midwife*, *Michelle*)

Michelle finds the discord between midwives and GPs challenging. Challenging communications and professional disputes creates potential for misunderstandings and undermines accessibility to supportive local networks. It seems there is nothing personal in this story; it is more about the discord of one profession with another. Many New Zealand GPs now lack the skills for providing obstetrics and avoid maternity [38]. The need to communicate across disciplines would appear important. Yet GPs in this practice simply do not recognise their responsibility to build relationships with midwives, until there is a client situation in which they need help. It seems not to occur to them that the midwives may also need their help from time to time, that a pre-established bond of trust would be in everybody’s interest. Working amongst differing ideologies can be emotionally exhausting [39]. Not addressing such discord at a national level gestures towards unsustainability and unrealistic unhealthy resilience.

### Intra-professional discord

Paula reveals that the issues of discord are also very real within the same profession:

*I would never say go out on your own. But there is an absolute lack of choice of who you work with around here. I think your back up has to be the most important thing. You absolutely must have professional support and regular time off. You have to give it to yourself. Even if you only got 2 births due this month*, *you just have to be away from that phone. You need a good strong practice partner relationship like I used to have. It*’*s almost like getting married actually. You know you*’*re seeing people at their rawest and when you*’*re tired and all the rest of it. That is the problem now. Around here you work really closely with people. So we have to have the same philosophy. I find it really difficult to back people up who think differently from me. The other LMC around here has her own issues. We have very different views on how things work. Her philosophy is quite different from mine. I do struggle with some of her decision making processes and I know that it would just wind me up if I had to work with her. So I*’*m quite happy to be here and she can call on me but I*’*m not going into a partnership. We could have joined together I suppose*, *it could have been lovely but it wouldn*’*t have been*; *I know it wouldn*’*t have been*. (*LMC midwife*, *Paula*)

Paula shows the significance of relationships in rural maternity experience but explains that just because there is another midwife in the community does not mean that the relationship between them will work. To work in a committed partnership in these isolated communities is “like a marriage”. As Paula explains, colleagues see each other at their most vulnerable, at times when they are raw. The level of trust needs to be accepting of the other ‘just as they are’. When the values are different, when the decisions of the other do not feel wise or safe, there is no foundation of trust on which to build. It feels safer to practice alone. In an urban setting the midwife could keep looking until she found another midwife with whom she felt a kindred spirit of safe practice. In the rural setting there are no other options.

When discord shows in rural maternity the taken for granted relationality of rural living reveals itself as that which matters most. Relationships matter personally and professionally. A Canadian study found that inter-professional tensions and interpersonal conflicts in rural regions only serve to exacerbate feelings of isolation [8]. Working together and appreciating each other’s unique contributions appears central to safe maternity care and positive experiences for all. The reassurance of being in relationship is in constant tension with the potential vulnerability of feeling discord and isolation. Not all rural maternity services are built on partnership and consensus within this current study. Stories of local integration and lack of local integration of services is evident.

The majority of the time, within the everyday rural lived experience, help from other(s) is available and locally accessible. Despite this confidence in local services maternity care providers and families can feel cut off from secondary services. This shows itself when things go wrong, when phone coverage is not there, when local help is not available and when local resources are insufficient to meet the need in that moment. Each health care professional and family is reliant on knowing others are ‘there’ and ‘accessible’. Physical and emotional isolation from others near and far can make rural living unsafe.

### Discord and unsafeness

When rural dwellers need help, when they are reliant on others, it is imperative that there are clear communications that appreciate the uniqueness of the local rural circumstances.

*I had a 34 week prem labourer*, *her second baby. She lived really remotely. It wasn*’*t far for me to get to*, *yet it was* ‘*getting the ambulance*’ ‘*telling the people at the secondary hospital*’. *Then the hospital staff ask*: “*Have you done swabs*?” “*Have you done this*?” “*Have you made sure of that*” “*Have you got her on the monitor*?” *It was 3 am in the morning I*’*m out in a really remote area in the women*’*s home and the baby is going to deliver*! “*No we haven*’*t got a monitor* - *I*’*ve got a sonicaid*!” *I just wanted paediatric back up and to get the baby to the hospital but it became a big argument between the ambulance service and the hospital about who would come and whether an incubator to retrieve the baby would be sent. The baby came out in relatively good condition*; *luckily I had a student midwife with me and another colleague that I used to work with. I waited 3 h as this messing around and argument was going on. The ambulance service was getting really frustrated and felt sorry for me. I just thought*, ‘*my god*!’ *The situation just hit me*. ‘*Thank god the baby is fine and the mum is fine*’. *We did all the things in this woman*’*s home and just got on with the job and did everything we could*, *keeping the baby warm*, *giving colostrum*, *doing blood sugars*. (*LMC midwife*, *Sally*)

Sally just had to do the best she could as the transportation drama unfolded on the phone. She is reliant on the help of others who are far. Unfortunately those far away do not appreciate the urgency of the situation. Once again living with discord is announced at a critical time in rural maternity care provision. Although it is not possible to determine whether the actions of the local midwife or the 3 h delay influenced the outcome for this infant the unnecessary added stress due to discord between services highlights vulnerability and Sally feeling unsafe.

In a moment of crisis Sally needed someone at the hospital to have a more perceptive understanding of the situation unfolding and relieve her of the burden of organising the plan so that she could attend to the reality in the ‘now’ in front of her. If decision making and risk reduction is to be optimised the interface of primary and secondary services needs to be courteous, tactful and focussed on the wellbeing of the women and baby.

## Discussion

Each maternity service and subsequent experience reflects the geographic, social, political region in which it has grown and the blend of skills locally available. Some qualities may seem constant yet they are never static and permanent. They are in constant play; day to day, person to person, region to region, and situation to situation. The qualities presented here do not have defined permanent boundaries, nor are they binary opposites of negative or positive, good or bad. On the contrary there is blurring within the tensions of what works and what constrains. That is the nature of human lived-experience. Acknowledging this plasticity reveals the continuous tensions at play within rural maternity.

This study reveals how rural communities are comprised of people in an interweaving tapestry of connectivity. Relationships matter in rural regions yet there are tensions at play within these relationships. Whilst congenial, supportive relationships are ideal, there were stories of breakdown. The data shows that when a relationship break down the consequences of poor communication is revealed as discord. Participants in this study were clear that functioning relationships with others in their community and those at the interface of secondary services were what made things work. The significance and meaning of relationships (personal and professional) becomes more apparent when absent or when discord is experienced. The limited choices as to whom one needs to rely on in small rural community may serve to magnify potential for discord. The importance of collegiality, or working together in a manner in which trust and mutual respect are built is vital in rural regions. Living with discord is feeling disharmony, unrest and unsettledness bringing a mood of anxiety into practice.

Relationships revealed themselves as essential to sustaining a sense of support. Heidegger tells us that we are never without others [26]. The rural maternity practitioner is always with others; if physically alone in a crisis the absence of help from another makes the aloneness even more stark. Relationships matter and are woven throughout maternity care [2, 34, 40, 41]. This is particularly pertinent in rural and remote regions when secondary services are far away. When working with others (far and near) is difficult or not possible, rural maternity experience is vulnerable. When relationships far and near are strong and reciprocal, safe maternity experience becomes safer and more enjoyable. Unfortunately it is evident in this study that not all relationships function as they should and could.

Notwithstanding personal differences there is always the tension that some relationships ‘happen of their own accord’ and others that must be ‘made to happen’ to keep practice safe and sustainable. For example, in the moment of pondering who to call for help, the ‘who’ is likely to sway the willingness to make such a call promptly. Anticipating a respectful response brings a very different mood to making such a decision as opposed to a sense that one will be judged, censured and belittled. A starting place to forging collegial relationships is understanding the need to get to know each other and thereafter offer support.

Within every story from participants, palpable moods emerged. Heidegger makes it clear that understandings unfold within moods. It is the moods of a particular place, national and regional politics, systems, persons and events that underpin our understanding of how we act within what is being experienced [26, 42, 43]. Heidegger describes how moods or attunements are not merely passing personal feelings or emotions but are intrinsically a part of the shared experience of Being-in-the-world. Attunements are thus not necessarily private [44]. Moods can be social and public and represent ways of Being-with-one-another. They provide a sense of collective and personal experience of how a situation is unfolding and how we are faring. A shared mood is therefore a public shared communication that reveals the situated experience of rural maternity. A mood of vulnerability, as expressed by several of the participants, permeates beyond the midwife herself; as, in contrast, does a mood of strong team support.

Pre-understandings provoke moods. When the discourse of one discipline is dis-respectful of another discipline (and vice versa) such pre-understandings go ahead of us shaping the moment of ‘now’ [28, 44]. Rural midwives work within their world, rural GPs within their world and hospital staff within theirs. Each professional group brings their own pre-understandings and mood to practice. Focussing on differences, the binary conflicts, issues of power and perceived subjection are important to acknowledge yet can be limiting and do not open to possibilities. Dichotomous thinking is a product of a society that thrives on arguments and wanting to win a position [45]. It infers that there are rigidly marked partitions in discordant voices with little recognition of a middle ground within which common meanings and significance can begin to be understood.

Sustainable rural maternity requires a focus and way of attuning that reaches beyond the confines of individuals and professional groups - this is not a new idea. Robertson argued for a more collaborative model in rural New Zealand maternity [46]. Divergent approaches arguably only weaken the strength and flexibility required in rural maternity relationships. Conversely, convergence of differing professional and personal approaches highlights the tensions within experiences and discloses what matters most.

Despite obvious differences something connects us in every moment. Exploring what matters most in rural maternity via multiple vantage points reveals that all involved want the same; safe positive outcomes. Each participant does what they do for the sake of what matters most to them. For Heidegger, the fundamental comportment of human beings to the world is care. How we enact that care is through concerned actions that discloses what matters most to us. The desire to care and actualise concerns into actions is an aspect of being-with others revealed through relationships. Participants find themselves in the world of rural maternity ‘concernfully’ dealing with what matters most to ‘them’. They trust that carrying out their unique professional responsibilities and their own set of concerns will achieve the best outcomes for mothers and infants in their communities. Yet it is the bringing of different voices to this interpretation that allows the diversity and commonality to be seen.

The importance of appreciating the differences in history of traditions cannot be underestimated. Gadamer tells us that differences in traditions can become understood more profoundly by appreciating pre-understandings and lead to a fusion of interpretive horizons previously unspoken [28]. Hunter and Segrott [47] showed how achieving midwifery professional autonomy through adoption of a model of care created defined boundaries between midwifery and obstetric colleagues leaving doctors excluded and undervalued. Similarly New Zealand midwives return to autonomy in 1990 resulted in a loss of GP control over primary maternity care [13] and continues to influence current professional relationships. As Gadamer [28] explains; ‘It is the tyranny of hidden prejudices [pre-understandings] that makes us deaf to what speaks to us in tradition’ (p. 272). Lampert [48] contends that it is our responsibility to make familiar that which seems alien and conversely make alien that which seems familiar. In this reflexive move it becomes possible to highlight and appreciate the distances between traditions (e.g. professional groups within maternity) and foster healthier relationships.

Coming to know and trust each other’s commitment to the client builds confidence between maternity care providers and enables a move forward to a place that works for everyone. This entails Midwives and GPs for example finding ways of working closely together that would be ‘unheard of’ in the urban areas. Drawing attention to our pre-understandings reveals what matters most, safe positive childbirth, and opens the possibility of an enjoyable community of rural maternity practice. Achieving safe positive childbirth experiences and resilient thriving rural communities is a possibility worth pursuing. This requires working co-operatively and not competitively, changing the mood across rural maternity services to one of trust and lessening any feelings of vulnerability. Exemplary communication and nurturing of collegial relationships is crucial.

There is a place in our shared human experience when knowing and understanding brings us into relationship with each other. When this occurs rural maternity care practitioners and maternity system decision makers attune to what matters most and nurture relationships that foster safe care for ‘this’ woman and ‘this’ baby; not personal, professional and organisational discord. Attuning to being-with-other starts with the gift of a helping hand, sharing of time and local resources, congenial conversations and a listening ear when needed. These simply ways of being-with build affable and congenial relationships that enable our concerns to translate into doing what needs to be done ‘now’ together. According to Heidegger it is not possible to verify attunements or moods but we can draw attention to them and awaken them [43]. The stories in this paper gesture a need to unpack what shapes pre-understandings to build such authentic open dialogue and awaken a mood of trust. All need to be heard to affect lasting optimal changes in these regions where a point of vulnerability can lead to catastrophic breakdown. People, whatever their role and wherever they live and work want to feel appreciated (loved), they need to feel understood and feel safe [49].

It is not possible to “make” everyone get on with everyone in their locality, but it is possible to reduce issues that cause much of the discord. When professional tensions are put aside to ask “what is the care this client needs and how best can we collectively provide such care?” it is likely that creative solutions that work in everyone’s best interests will be found. Recognition of the need to attune collectedly, of being-with-others in ways that enable trusting connections that traverse differences and nurtures reciprocal understandings is essential.

It is vital not to view rural maternity as inherently problematic. Rural maternity globally is a dynamic environment that is often reported as innovative [50]. Despite myriad challenges rural maternity providers are continually reported as providing safe maternity care [51]; this is no less true in New Zealand [23]. Nevertheless this study uncovers struggles in relationships that challenge sustainable, safe and integrative rural maternity services.

At times it appears that rural practitioners carry the weight of responsibility alone; held within the confines of a single professional group culture. False dichotomies, battles, conflicts, territorial disputes and boundary protection strategies appear to impede the quality improvements for individual and community. Good communication and collegial rapport as described above are key to ensuring integrated maternity services that maintain the safety of mothers and babies between remote and secondary urban services [52, 53]. Hierarchical structures that dictate how relationships are allowed to happen are not useful and can impede progress. Each individual and collective experience needs valuing [54]. Struggling relationships in rural regions due to the constant need for negotiating myriad complexities, social influences and risks inherent in rural regions has been identified previously [55]. Collaboration and cooperation is thus needed whilst acknowledging professional agendas, local risks and complexities.

All maternity care providers, whatever their discipline, are reliant on others. Respectful collegial working relationships at the interface of services and mutual appreciation of each other’s context, skills and expertise are vital. The rural maternity experience is reliant on clear open professional and friendly communications with others across maternity health care teams despite differences in location and ways of working. In one region in this study ambulance crew and GPs met regularly to discuss recent transfers and how they were managed. It was unfortunate that the local rural midwives were not included. It was not clear why this was the case. Inviting local midwives into multi-disciplinary meetings would be one simple initiative that promotes collaborative learning, debriefing and support.

Fostering better collaborative learning in remote rural regions with GPs, midwives, nurses and ambulance crew would be advantageous. Inter-professional learning opportunities are recommended as a means to build feelings of credibility and confidence in each other’s roles [56]. Sharing such events may break down feelings of discord and help with collaboration across sectors. It has been found that such collaborative workshops develop appreciation of others’ skills and areas of responsibilities and allow the possibility of cohesive common understandings and contextual meanings [56]. This process would engender transformative learning and a reflexivity that brings understanding of what it would be to stand in each other’s shoes. Although such initiatives remain rare [57], they are worthy of exploration and could facilitate more cohesive communities of rural practice.

### Strengths and limitations

This is a small New Zealand study and is focussed on producing findings that are not generalisable, but are potentially transferable. The context of the study is a well-resourced high income region and acknowledges the different range of complexities in low to middle income countries. Achieving trustworthiness and ensuring conclusions were plausible and credible was essential in maintaining integrity and dependability of this study [58]. The study is balanced through integration of human voices and philosophical notions. The chosen methodology enabled the voices of those living in, and living through, the daily contextual realities of New Zealand rural maternity to be heard. This permits discussion and recommendations that are derived from the lived experiences of the phenomenon. There are always more voices and further interpretations. For example the voices of core staff receiving rural mothers and their maternity health care providers on transfer would be valuable. Likewise the voices of family members of health care providers and mothers are absent from this study. Openness was demonstrated through the use of a detailed audit trail and concreteness was established through presentation of pre-understandings and justification of the study. Resonance has been shown through sharing and discussing findings providing reassurance of plausibility and the study has been actualised through acceptance for peer reviewed publications and presentations. In addition the study and subsequent report has been checked against the COREQ reporting framework [59].

Hermeneutic phenomenology acknowledges that there is no final truth but a pointing to what is happening. There is always more to interpret from the data in any study of this nature; we offer our plausible interpretations providing opportunity for a way forward into further research, thinking and practice development. Although there is safety in a formulaic recipe to fix things it must always be contingent on the flux of practice reality that is lived in and lived through.

## Conclusion

Relationships founded on mutual understanding and attuned to trust matter in rural maternity. They keep childbirth safe and enable maternity care providers to work sustainably. Hidden unspoken pre-understandings of individuals and groups create struggle and tension in relationships leading to discord. Discord between rural practitioners is unsustainable and harmful. When our unique historical prejudices are uncovered what matters most in life becomes clearer and trust can awaken between us. Attuning to trust amongst rural dwellers and far away service providers helps keep rural childbirth safe. Trust builds resonate rural communities of practice within which everyone can flourish, feel accepted, supported and safe.
